# Stable H-O Isotopic Composition and Water Quality Assessment of Surface Water and Groundwater: A Case Study in the Dabie Mountains, Central China

**DOI:** 10.3390/ijerph16214076

**Published:** 2019-10-23

**Authors:** Kunhua Yang, Guilin Han, Chao Song, Peng Zhang

**Affiliations:** 1 Institute of Earth Sciences, China University of Geosciences, Beijing 100083, China; kunhuayang@cugb.edu.cn (K.Y.); Zpeng@cugb.edu.cn (P.Z.); 2 Institute of Hydrogeology and Environmental Geology, Chinese Academy of Geological Sciences, Shijiazhuang 050061, China; songchao@mail.cgs.gov.cn

**Keywords:** hydrogen isotope, oxygen isotope, water quality assessment, surface water, groundwater, Central China

## Abstract

In order to understand the water cycle and assess the water quality for irrigation purposes in the Upper Pi River Basin (UPRB), which is the northern slope of the Dabie Mountains, 68 surface water and groundwater samples were collected and analyzed for H-O isotopes and hydrochemistry during the high-flow season in 2017 and 2018. The results show that ranges of hydrogen and oxygen isotopic composition (δ^2^H: −68.8‰ to −40.8‰, δ^18^O: −10.05‰ to −5.05‰) are controlled by the medium latitude and high altitude of the UPRB. Among different types of water, the δ^2^H and δ^18^O values can be ordered as follows: reservoir water < spring water ≈ river water < pond water. The water of the upstream medium and small reservoir is enriched with lighter isotopes that is likely related to more exchange with rainwater and less residence time; however, large reservoirs are similar to the upstream river and spring in terms of the H-O isotopic composition. Hydro-chemical facies are dominated by the Ca-HCO_3_ type in the UPRB, which reflects fresh recharged water from rainfall, and few samples are of the Ca-Cl type that is caused by intensive evaporation. The water quality for irrigation purposes was also evaluated. According to the Wilcox diagram, United States Salinity Laboratory (USSL) diagram, magnesium hazard, and Kelly’s ratio, all water samples have been considered suitable for irrigation water.

## 1. Introduction

In mountainous areas, climate change and human activities can have a thorough impact on the water cycle, such as increased precipitation, evapotranspiration, and the consumption of surface water and groundwater [1,2,3]. Furthermore, these changes in the meteoric water can influence biogeochemical cycles through water flow [4]. To better forecast potential changes in the climate and ecological system, an understanding of the hydrological behavior is required [5,6]. The Pi River originates in the Dabie Mountains, Central China, and the Upper Pi River basin (UPRB) frequently exhibits extreme precipitation owing to complex terrain and climate change [7]. Rainstorm-induced mountain flooding causes huge economic losses and threatens human life in this region. The main sources of the river water generally include recent precipitation and groundwater recharge [8]. However, catchment-based studies of surface water and groundwater in the UPRB are less numerous, despite the fact that it is significantly important for reducing the risk of flooding disasters and understanding the impacts of climate change on the water cycle. On the other hand, the Pi River is the main water source for a 7750 km^2^ irrigation area and about 13 million people [9], and four large reservoirs in the UPRB are important water conservancy facilities for ensuring that there is enough water for drinking, domestic, agricultural, and industrial purposes. Therefore, monitoring of the water quality in the UPRB is important.

Stable isotopes (δ^2^H, δ^18^O) in meteoric water are able to trace the water source and understand hydrological processes and the water cycle [10,11,12]. Variation in the stable isotopic composition of river water may be able to identify source water dynamics, because the isotopic composition of the river water is mainly connected to local precipitation [8,13], temperature [14], and snowmelt/groundwater recharge [15,16]. On the other hand, the stable isotopic composition of river water can reflect the important influence of anthropogenic processes like damming and water storage [17]. In order to improve understanding of the water cycle in terrestrial systems, δ^2^H and δ^18^O are monitored to analyze isoscapes (i.e., the spatial-temporal isotope distribution) across the world [18,19,20]. In the 1970s, the Global Network of Isotopes in Precipitation (GNIP), initiated by the International Atomic Energy Agency (IAEA) and the World Meteorological Organization (WMO), focused on an observation network of stable hydrogen and oxygen isotope data of precipitation [21]. In the 2000s, to complement the GNIP, IAEA launched the Global Network of Isotopes in Rivers (GNIR), which collects isotope data of surface water around the world [13]. On a local scale, seasonal isotopic patterns within river water can reflect changes in the mean elevation of a river’s source water [22]. The method of δ^2^H and δ^18^O based on the Rayleigh distillation equation and mass conservation is used to quantify the evaporation and recharging of a river [23]. Moreover, the deuterium excess calculation demonstrates that land use has an important impact on the hydrologic cycle in a watershed [24]. On the other hand, the temporal distribution of stable oxygen isotopes in the Changjiang river water indicates the time lag of river water responding to meteoric precipitation, and is the result of increasing trapping and water regulation effects of numerous dams [25]. These surveys have indeed proven the values of stable hydrogen and oxygen isotopes in hydrological studies. As a result, the stable H and O isotope technique is a potentially powerful tool for the monitoring of eco-hydrological systems [26].

To investigate the hydrological processes in the UPRB, including the precipitation, the interaction between surface water and groundwater, and the evaporation, we examine the spatial variation of the stable hydrogen and oxygen isotopic composition of surface water and groundwater in the high-flow season. Additionally, we assess the water quality for irrigation purposes based on the hydrochemistry of surface and groundwater in the UPRB. The results of this study are expected to provide more evidence on the water environment and quality assessment for irrigation purposes in the UPRB, which can present reasonable strategies for water management in the locality.

## 2. Materials and Methods

### 2.1. Background of the Study Area

#### 2.1.1. Location

**Figure 1:**
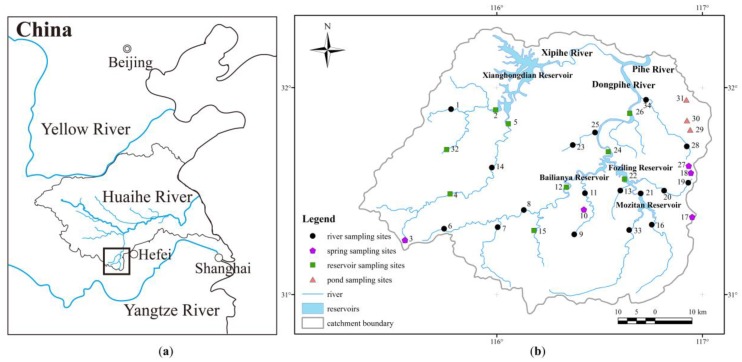
(**a**) Location of the Upper Pi River basin (UPRB) and (**b**) water sampling sites.

The study area is situated in Anhui Province, Central China (115°66′~116°53′ E, 30°95′~31°65′ N), and covers a surface area of approximately 4350 km^2^ (Figure 1a). It is located on the northern slope of the Dabie Mountains, with a maximum altitude of 1750 m. The altitude is higher in the southwest and generally decreases northeastward. The main types of land uses are forest (65%), grassland (27%), farmland (6%), and water area (1%) [27]. Two rivers, the East Pi River and West Pi River, flow northeastward through the study area, and they are the headwaters of the Pi River, a southern branch of the Huaihe River (Figure 1b). During flood events, a peak discharge of up to 5600 m^3^/s has been recorded at the Hengpaitou hydrologic station [28], where the West Pi River and East Pi River join the main stream of the Pi River.

#### 2.1.2. Climate

This region is located in the transitional zone between the abundant rainfall area of Southern China and the arid area of Northern China [7]. The predominant climate is semi-humid monsoon conditions. Coupled with undulating terrain and relatively high altitude, rainstorms and flooding events frequently occur in this area in the high-flow season (from June to October). From 1968 to 2018, the mean annual air temperature was 15.5 °C, and the mean minimum and maximum air temperature was 11.4 °C and 21.0 °C, respectively [29]. The annual rainfall is around 730–2010 mm, of which 67% occurs during the period from June to September [30].

#### 2.1.3. Reservoirs

In the study area, there are four large reservoirs (storage capacity > 0.1 km^3^), which are an important water source for approximately 13 million people: Mozitan Reservoir, Bailianya Reservoir, Foziling Reservoir, and Xianghongdian Reservoir. Among them, Mozitan Reservoir, Bailianya Reservoir, and Foziling Reservoir are situated in the East Pi River, and Xianghongdian Reservoir is situated in the West Pi River. The construction of large reservoirs is conducted with the aim of providing multiple benefits, including a water supply and flood control, in the study area [31]. Besides, there are many small and middle reservoirs, such as the Banjiezhuizi Reservoir in the lower reaches of the East Pi River.

### 2.2. Sampling Processes

Two series of water were sampled in the high-flow season (October 2017 and September 2018, respectively). During each sampling period, 29 surface water samples and 5 groundwater samples were collected, including 17 river water samples, 9 reservoir water samples, 3 pond water samples, and 5 spring water samples (Figure 1b). The water sampled from the river, reservoir, and pond was generally taken from the bank at a sampling depth of ca. 50 cm. The groundwater was collected from creeks where mountain springs flow out. Before sampling in the field, the high density polyethylene (HDPE) bottles were cleaned using detergent, and were then totally soaked in deionized water for six hours and finally dried out in an electric oven. At the sampling sites, we used a multiparameter instrument (Pro Plus, YSI Inc./Xylem Inc., Yellow Springs, OH, USA) to determine the total dissolved solid (TDS) and electrical conductivity (EC), and we used GPS to record the longitude, latitude, and altitude. In the field, every water sample was filtered using a 0.22 μm cellulose acetate membrane syringe filter and transferred to a pre-cleaned PET (polyethylene terephthalate) bottle. Next, the sample bottle was sealed using Parafilm ^®^ M film and stored in a refrigerator at about 4 °C.

### 2.3. Major Cations and Anions

Concentrations of major cations (Na^+^, K^+^, Ca^2+^, and Mg^2+^) were measured by ICP-OES (ICAP 6300, Thermo Scientific Inc., Rochester, NY, USA) with a precision of ±3% at the Institute of Hydrogeology and Environmental Geology, Chinese Academy of Geological Sciences. Volumetric titration with Disodium Ethylene diamine tetra acetate (EDTA, 0.01N) with an analytical error of <2% was used to analyze SO_4_^2−^. The titration method was employed to analyze Cl^−^ and HCO_3_^−^. An ultraviolet-visible (UV-VIS) spectrophotometer (UV-1780, Shimadzu, Japan) was used to analyze NO_3_^−^. The charge balance error was calculated to validate the quality of major ion analysis, which was within ±5%.

### 2.4. Isotope Analysis

The analysis for δ^2^H and δ^18^O was conducted at the Institute of Geographic Sciences and Natural Resources Research, Chinese Academy of Sciences, and the equipment included Triple-Isotopic Water Analyzers (Model TIWA-45-EP, Los Gatos Research Inc., San Jose, CA, USA) with laser spectroscopy techniques. In order to monitor the data quality, one isotopic standard was measured for every three samples. Each sample/standard was analyzed six times, and the first two results were discarded for avoiding memory effects. The final result of each sample/standard was the average of the last four results. The measurement results of hydrogen and oxygen isotopes were expressed as follows:

δ^2^H (‰) = [(^2^H/^1^H)*_sample_*/(^2^H/^1^H)*_standard_* − 1] × 10^3^ (1)

δ^18^O (‰) = [(^18^O/^16^O)*_sample_*/(^18^O/^16^O)*_standard_* − 1] × 10^3^ (2)

The isotope data are reported in per mill (‰) relative to the standard Vienna Standard Mean Ocean Water (V-SMOW), and the measurement precision was ±0.5‰ (1σ) for δ^2^H and ±0.1‰ (1σ) for δ^18^O.

## 3. Results and Discussion

### 3.1. Stable H-O Isotopic Geochemistry

#### 3.1.1. Hydrogen and Oxygen Isotopic Composition

**Figure 2:**
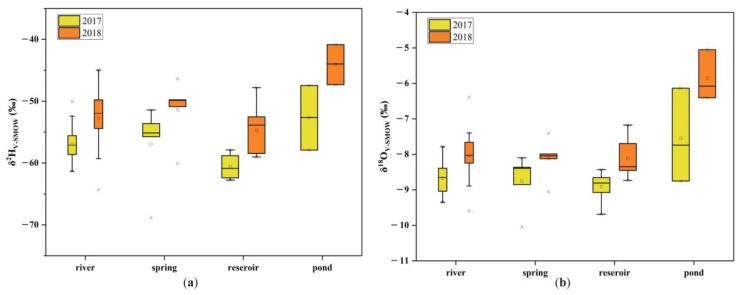
Box diagrams of (**a**) δ^2^H and (**b**) δ^18^O values in different water bodies.

In general, the δ^2^H and δ^18^O values can be ordered as follows: reservoir water < spring water ≈ river water < pond water (Figure 2 and Table 1). The distribution of δ^2^H and δ^18^O in different water bodies was similar in 2017 and 2018, although the water samples were more enriched with ^2^H and ^18^O in 2018. It is possible that more isotopic fractionation occurred in September 2018 due to a warmer and drier environment, i.e., a higher temperature and lower humidity (Table 2). However, the standard deviation of all water samples between 2017 and 2018 was only 6.7% for δ^2^H and 8.2% for δ^18^O. Hence, the deviation was considered acceptable.

The relation between δ^2^H and δ^18^O of water samples with the Global Meteoric Water Line (GMWL: Equation (3)) [32] and Local Meteoric Water Line of Nanjing (LMWL-Nanjing: Equation (4) [21]) is presented in Figure 2.

δ^2^H = 8 × δ^18^O + 10 (3)

δ^2^H = 8.43 (±0.18) × δ^18^O + 15.89 (±1.54) (n = 58, r^2^ = 0.98, regression type: PWLSR) (4)

LMWL-Nanjing was obtained from the Global Network of Isotope in Precipitation (GNIP) [21] and was collected from Nanjing City, which is only 250 km away from the study area.

**Figure 3:**
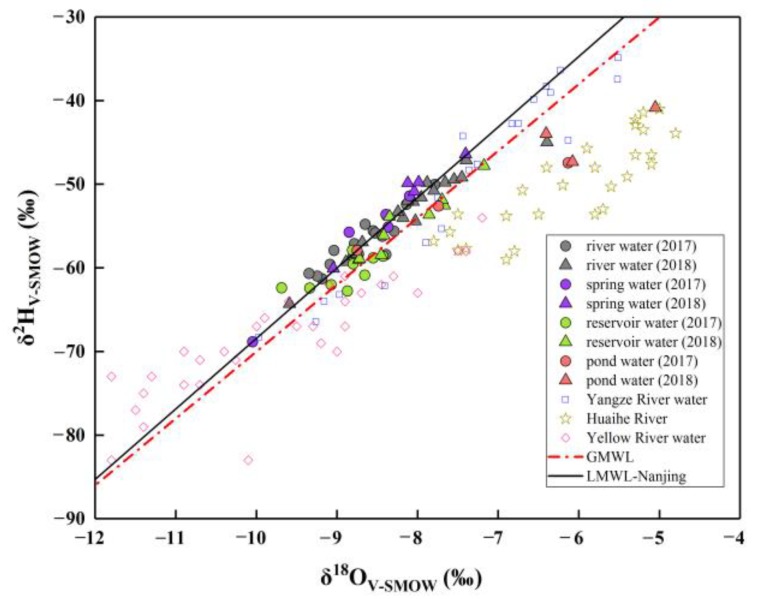
Scatterplots of δ^2^H and δ^18^O in the UPRB, the Yangtze River [33], the Huaihe River [20], and the Yellow River [34], and the correlation with the Global Meteoric Water Line (GWML) [32] and Local Meteoric Water Line of Nanjing (LMWL-Nanjing) [21].

It can be seen from Figure 3 that most of the water samples in the study area lie on or fall near the GMWL and LMWL-Nanjing, except for several pond water samples in the plain area. Meanwhile, in Figure 3, water samples in the UPRB are located in a region where the time-series isotopic composition of the Yangtze River water (Datong station) [33] and spatiotemporal-series isotopic composition of the Yellow River [34] partly coincide. On the other hand, we have also displayed the stable isotope data in the Huaihe River, where the UPRB is the headwater area of the sub-basin. Nevertheless, Huaihe River water samples significantly deviate from the GMWL, the LMWL-Nanjing, and the points of the UPRB. This phenomenon is because most samples in the Huaihe River were collected from the plain or swale. In other words, water bodies in the low-elevation area show larger isotopic fractionation than those in the mountainous environment, which is related to kinetic fractionation that occurs during the evaporation processes.

Differences in the isotopic composition suggest that it is responsible for different hydrological processes. The statistical δ^2^H and δ^18^O values of different types of water sample are shown in Table 3. For river water, overall, the mean values in 2017 show no significant deviation from those in 2018; however, the δ^2^H and δ^18^O of the river water exhibit a wider range in 2018 than in 2017. The mean values of δ^2^H and δ^18^O are similar for spring water and river water, both in 2017 and 2018. Compared with river water and spring water, reservoir water is more enriched with ^1^H and ^16^O, and its δ^2^H and δ^18^O ranges are narrower. Among the nine reservoir water samples, five samples were collected from medium and small reservoirs (No. 4, 5, 15, 26, and 32), and the other four were collected from large reservoirs (No. 2, 12, 22, and 24). For the reservoir water sampled from the upstream area of the large reservoir (including No. 12, because it is in the mouth area where the river water flow in), δ^2^H and δ^18^O are lower, for which the average of δ^2^H is −62.1‰ in 2017 and −56.1‰ in 2018; for the remaining reservoir water (i.e., No. 2, 22, 24, and 26), the average of δ^2^H is −58.7‰ in 2017 and −52.9‰ in 2018. Apparently, the isotopic composition of the former is lighter than that of the latter. The δ^2^H and δ^18^O of pond water are obviously higher than those of river water, spring water, and reservoir water.

Therefore, the samples were divided into three groups, according to the stable isotopic composition: the water in medium and small reservoirs, the pond water, and the remaining water. The pond water is most enriched with heavy isotopes, which indicates that it is likely related to significant evaporation. On the contrary, the water in medium and small reservoirs is mostly enriched with light isotopes, which is probably due to rapid recharging from rainwater. On the other hand, similar values for river water and spring water prove that the two are consistent in terms of hydrological behavior, and the slightly heavier isotopic composition of river and spring water demonstrates higher evaporation than the water in medium and small reservoirs. Moreover, large reservoirs mainly receive water from rivers. Therefore, the water in large reservoirs is similar to river and spring water in terms of stable isotopes.

#### 3.1.2. Impact of Water–Rock Interaction and Evaporation

**Figure 4:**
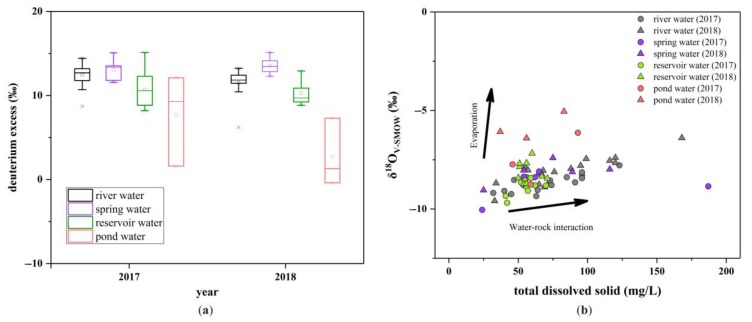
(**a**) Deuterium excess (*d*-excess) values of different types of water samples in the study area. (**b**) Total dissolved solid (TDS) versus δ^18^O of water samples.

Deuterium excess (*d*-excess), defined as *d*-excess = δ^2^H − 8 × δ^18^O [35], is a dual isotope index and has been widely applied to precipitation and continental water studies [36,37,38]. The global average *d*-excess of meteoric water is 10‰ [32]. However, the *d*-excess of surface water and groundwater is influenced by the moisture source of local precipitation, evaporation, water–rock interaction, and recharging processes [11,17,39]. In the study area, overall, the *d*-excess values can be ordered as follows: spring water > river water > reservoir water > pond water (Figure 4a). The sequences for 2017 and 2018 are the same, which indicates that the hydrological process has not been influenced by extreme events. However, the variability in *d*-excess values of different water bodies was higher in 2017.

With a total surface area of only 4350 km^2^, the moisture source is not regarded as the main factor in the variation of *d*-excess of different types of water. To discriminate between water–rock interaction and evaporation processes, the scatterplots of TDS versus δ^18^O of different water bodies are illustrated in Figure 4b, and distinction is obvious among the four water bodies. It is clear that river water and spring water have higher TDS values, and pond water has higher δ^18^O values. Chemical weathering in water–rock interaction can increase the TDS in water [40]. Combined with a subtropical climate and siliceous-predominant rock in the study area, the humid and warm environment contributes to chemical weathering, and subsequently, the dissolved matter is carried by flowing water. Therefore, river water and spring water have higher TDS values than other water in the study area. However, large reservoirs mainly receive rainwater and river drainage from high-elevation areas, and the semi-closed water bodies, such as the pond, exchange less substances with the environment relative to the river. Hence, reservoir water and pond water have lower TDS values. In addition, due to the wider surface area of the reservoir and pond, the water experiences more intensive evaporation and had higher δ^18^O values, especially in the pond. In sum, river water and spring water are significantly affected by water–rock interactions, while pond water experiences more evaporation. 

#### 3.1.3. Isotopic Distribution in the Upper Pi River Basin

**Figure 5:**
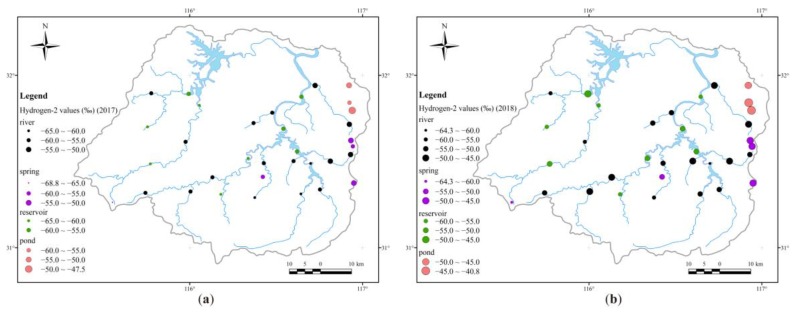
Spatial distribution of δ^2^H values in the Upper Pi River basin in (**a**) 2017 and (**b**) 2018.

Figure 5 shows the spatial variation of δ^2^H values in different water samples in the UPRB. The δ^18^O values show a similar distribution to δ^2^H values, so we have only illustrated the distribution of hydrogen isotopes. Spatially, the hydrogen-18 isotope shows greater enrichment in the east and is more depleted in the west. This is probably due to the relatively lower altitude in the East Pi River basin. In other words, in the low altitude area, the air temperature is higher and humidity is lower, which causes higher evaporation at the water’s surface and contributes to the heavier stable hydrogen and oxygen isotopic composition. On the other hand, in the eastern part of the basin, the average δ^2^H value (−53.6‰ in 2017 and −48.7‰ in 2018) of the three spring water samples (No. 17, 18, and 27) is close to that (−53.6‰ in 2017 and −49.0‰ in 2018) of the upper stream samples (No. 19, 20, and 28), which proves that the two frequently transformed. According to the similarly higher δ^2^H and δ^18^O values, the eastern part of Foziling Reservoir is mainly influenced by the Shiyang Stream (No. 11) and the Bailianya Reservoir (No. 12) instead of the drainage from bottom water in the Mozitan Reservoir (No. 21) (Figure 5), which has lower δ^2^H and δ^18^O values that may be caused by thermal stratification in the long water residence time [41].

**Figure 6:**
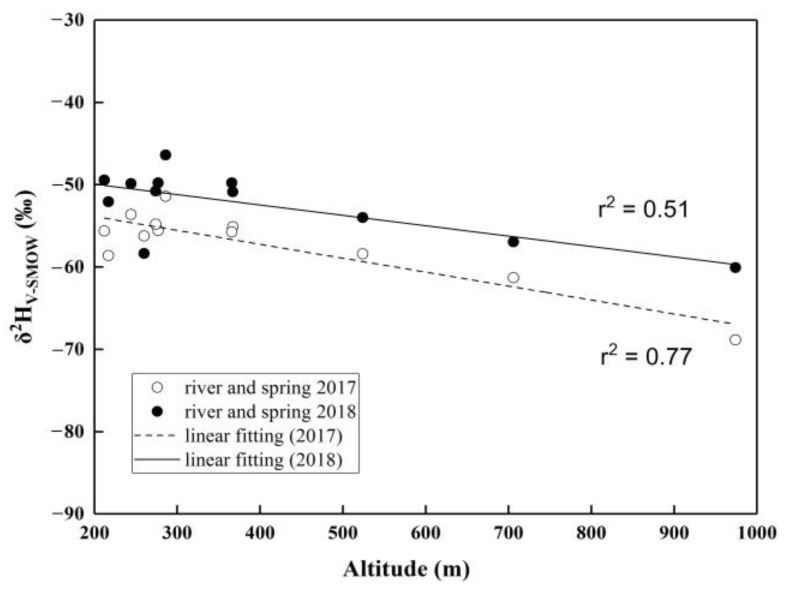
Correlation between δ^2^H and altitude (above 200 m) of river water and spring water.

The “altitude effect” reflects the linear relationship between the change in the isotopic composition of precipitation and change in elevation in the mountainous area. In high-elevation areas, the “altitude effect” of the stable hydrogen and oxygen isotopic composition in precipitation can be directly reflected by river water [42]. Subsequently, in the catchment which is mainly recharged by precipitation, surface water also shows a corresponding relationship between the isotopic composition and altitude. In terms of the negative correlation between the isotopic composition and altitude, river water and spring water located at an elevation above 200 m were selected to examine the “altitude effect” that is caused by rainfall (Figure 6). Reservoir water and pond water were removed, because too much evaporation causes isotopic fractionation. A glance at Figure 6 reveals the good linear fitting between δ^2^H and the altitude of river water and spring water in the mountainous area, and the negative correlation indicates that the river and spring are dependent on rainfall, especially in the area with an elevation of more than 300 m. The relationships between δ^2^H and altitude (A) in October 2017 (Equation (5)) and September 2018 (Equation (6)) are as follows:

δ^2^H (‰) = −0.0169A − 50.4737 (r^2^ = 0.77) (5)

δ^2^H (‰) = −0.0126A − 47.4177 (r^2^ = 0.51) (6)

This means that the hydrogen isotopic composition of river water and spring water decreases linearly with increasing elevation by at least 1.26‰/100 m in the UPRB at an elevation above 200 m. Moreover, this degree of decreasing δ^2^H may be intensified with a decreasing temperature and increasing humidify and rainfall amount.

### 3.2. Assessment of Water Quality for Irrigation Purposes

The study area has abundant water resources, including four large reservoirs and two main rivers, which are the main suppliers for the Pihe irrigation area of 7750 km^2^ and drinking purposes for about 13 million people [9]. Therefore, it is significant to assess the water quality of surface water and groundwater in the Upper Pi River Basin.

#### 3.2.1. Hydro-Chemical Classification

The hydrochemistry of surface water and groundwater is the main factor that determines its suitability for irrigation [43,44]. Different compositions of ions can be used to identify potential hydro-chemical origins based on standard Piper plots [45]. Therefore, the spatial variation of hydro-chemical classification is useful for understanding the relationship and evolution of surface and groundwater [46].

**Figure 7:**
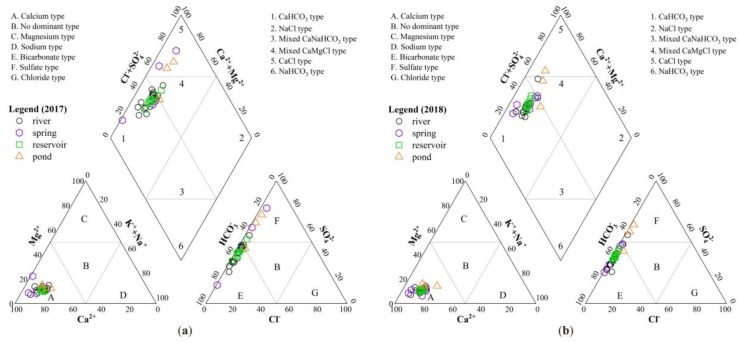
The piper diagram of the surface water and groundwater in (**a**) 2017 and (**b**) 2018.

The Piper diagram of surface water and groundwater in 2017 and 2018 was drawn by Origin 9.0 software (Figure 7a,b). Most water samples fall in the Ca-HCO_3_ type (zone 1), reflecting rainwater as the main recharging source. Only several samples fall in the Ca-Cl type (zone 5) and Ca-Mg-Cl type (zone 4). The results suggest that Ca-HCO_3_ is the dominant hydro-chemical facies for surface water and groundwater in the Upper Pi River Basin. Besides, the appearance of Ca-Cl hydro-chemical facies shows more marked salinization in the pond, which is likely caused by intensive evaporation.

#### 3.2.2. Water Quality Assessment

To examine the water quality and its suitability for irrigation purposes [47,48,49], several salinity indices, including the soluble sodium percentage (SSP), sodium absorption ratio (SAR), magnesium hazard (MH), and Kelly’s ratio (KR), were calculated:

SSP = Na^+^ × 100/[Ca^2+^ + Mg^2+^ + Na^+^ + K^+^] (7)

SAR = Na^+^/[(Ca^2+^ + Mg^2+^)/2]^0.5^ (8)

MH = Mg^2+^ × 100/(Ca^2+^ + Mg^2+^) (9)

KR = Na^+^/(Ca^2+^ + Mg^2+^) (10)

where all ions are expressed in the milliequivalent per liter (meq/L). The EC values and computed value of Na%, SAR, MH, and KR in the UPRB are presented in Table 4.

**Figure 8:**
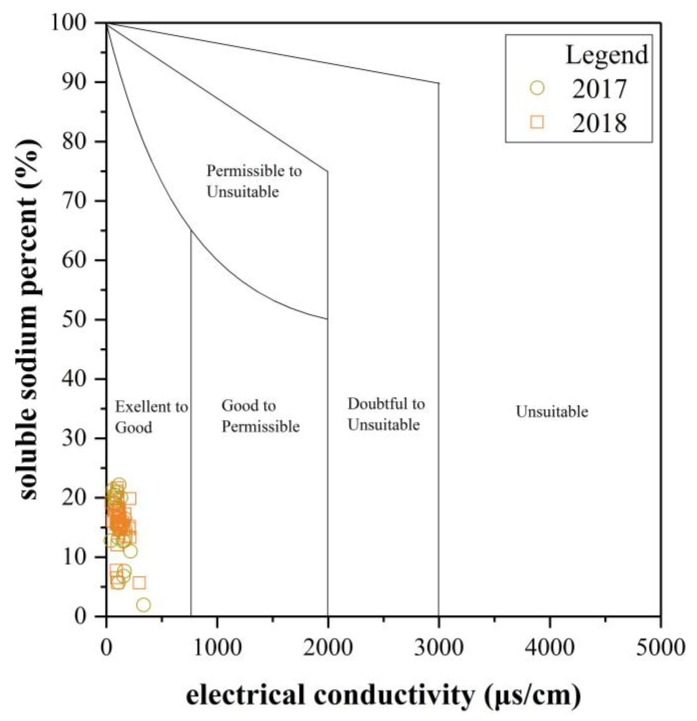
Wilcox diagram for evaluating the suitability of water for irrigation [50].

The soluble sodium percent (SSP) is a common parameter used to assess water’s suitability for irrigational purposes [50], denoted as Equation (7). The sodium in the water can displace the calcium and magnesium in the soil, causing a decrease in the ability of the soil to form stable aggregates and a loss of the soil structure. Irrigation water with a high sodium percentage can reduce the permeability of the soil, which consequently decreases the internal drainage of the soil and eventually affects plant growth [51]. Values of SSP < 50 indicate that water is suitable for irrigation, while SSP > 50 is considered unsafe for irrigation [50]. In the UPRB, SSP values of surface water and groundwater samples range from 1.95 to 22.18. Furthermore, according to the Wilcox diagram (Figure 8), all samples lie in the excellent to good zone.

**Figure 9:**
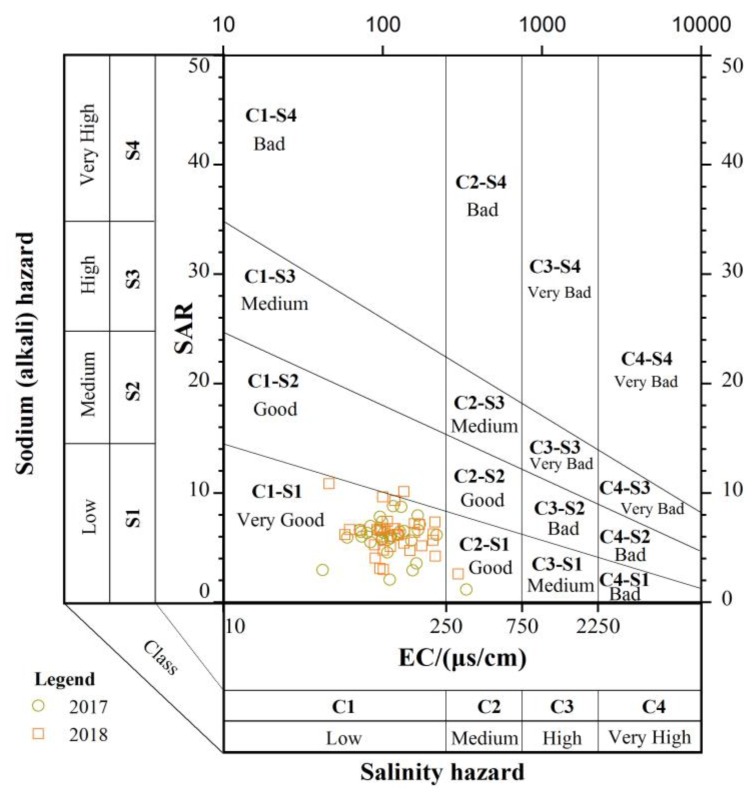
United States Salinity Laboratory (USSL) diagram for the classification of irrigation water (after the United States Salinity Laboratory [53]).

Additionally, because the sodium concentration can reduce the soil permeability and soil structure, the sodium adsorption ratio (SAR) is used to evaluate the suitability of water for use in agricultural irrigation [52]. Hence, a United States Salinity Laboratory (USSL) diagram (Figure 9) was employed to classify irrigation water. The EC value of the X-axis represents the salinity hazard, and the SAR value of the Y-axis represents the sodium (alkali) hazard. Based on the EC, irrigation water can be classified into four categories (EC < 250 μs/cm: Low-salinity water; 250 < EC < 750 μs/cm: Medium-salinity water; 750 < EC < 2250 μs/cm: High-salinity water; EC > 2250 μs/cm: Very high salinity water). Furthermore, a USSL diagram based on SAR can be divided into four categories. In this study, the USSL diagram shows that most water samples are found within the range of the very good category, except for three samples, which fall in the good category. Therefore, surface water and groundwater in the UPRB are suitable for use as irrigation water.

Calcium and magnesium maintain a state of equilibrium in most water. However, a high magnesium content makes soil become more alkaline, which has an adverse effect on the crop yield. Accordingly, a ratio, namely the index of magnesium hazard, was developed [54,55]. An MH value of less than 50 is considered suitable for irrigation. In the UPRB, all of the samples collected showed an MH ratio < 50% (suitable for irrigation).

Kelly’s ratio (KR) is another important indicator for the evaluation of water for agricultural suitability proposed in [56]. A Kelly’s ratio value greater than 1 indicates an excess level of sodium in water, while a value lower than 1 for Kelly’s ratio is considered suitable for irrigation [57]. In this research, all water samples fall in the suitable range for irrigation purposes (KR < 1).

### 3.3. Instructions for Local Water Management

In this study, the ultimate aim was to provide reasonable water management strategies in the UPRB. The isotope results show that the river and spring water are directly derived from rainfall and are frequently transformed, especially in the area at an elevation of above 300 m. However, large reservoirs are constructed in the area below 150 m, which mainly receives water from the rivers. Therefore, for more effective flood protection, hydrological monitoring of the rivers should be conducted in the area with an elevation of 300 m. Additionally, the research demonstrates that river, spring, reservoir water, and rainfall are closely interconnected, and these water sources are suitable for irrigation purposes. However, the river and spring water are obviously affected by water–rock interactions. Therefore, in the next step, geology-based hydrological studies in the UPRB will be beneficial for examining the source of heavy metals and harmful elements in the surface water and groundwater. Considering transformation between river water and spring water, the prevention of pollution in river water can ensure sustainability of the groundwater supply, especially in the upstream areas.

## 4. Conclusions

In the Upper Pi River basin (UPRB), surface water and groundwater are mainly recharged by rainwater in the high-flow season, which refers to the abundant rainfall resource in the mountainous area. Overall, the spatial distribution of hydrogen and oxygen isotopes reflects the separation of different water bodies. Medium and small reservoir water has the lowest δ^2^H and δ^18^O values, spring water is similar to river water in terms of the H-O isotopic composition, pond water has the heaviest isotopic composition, and H-O isotopic data of large reservoir water shows the mixing of river/spring water and rainwater. Furthermore, the similar isotopic composition and TDS proves that river water and spring water are rapidly transformed in upstream areas, and are obviously affected by water–rock interactions. Moreover, the good linear relationship between δ^2^H and the altitude of river water and spring water indicates that they are strongly dependent on direct recharging from rainfall in mountainous areas with an elevation of more than 300 m. The water in large reservoirs mixes with surface water, groundwater, and rainwater, whereas the water of medium and small reservoirs is dominated by rainwater. Pond water is evaporated clearly according to stable isotopes and hydro-chemical facies, which show impacts of human activities on the water cycle. Hence, the stable hydrogen and oxygen isotopes have demonstrated the potential to trace the water cycle in the UPRB. On the other hand, water quality diagrams, including the Wilcox diagram and USSL diagram, and other indices demonstrate that surface water and groundwater in the UPRB are considered suitable for irrigation water. In sum, water is rapidly transformed among the rainfall, rivers, springs, and medium and small reservoirs, whereas large reservoirs can increase the resident time of the water and matters. Investigating the H-O isotopic variability of surface water and groundwater in the UPRB could help in better understanding the materials cycle in the riverine studies in the future.

## Figures and Tables

**Table 1:** Locations and isotopic compositions of river, spring, reservoir, and pond water samples.

Water Types	No.	Stream	Log. °E	Lat. °N	Alt. m	δ^2^H	δ^18^O	*d*-Excess	δ^2^H	δ^18^O	*d*-Excess
						‰	‰	‰	‰	‰	‰
						2017			2018		
River water	1	Xipi	115.89	31.45	146	−58.4	−8.73	11.5	−59.3	−8.89	11.8
	6	Manshuim	115.87	31.16	524	−58.4	−8.39	8.7	−54.0	−8.18	11.5
	7	Wugui	116.00	31.16	277	−55.6	−8.55	12.8	−49.8	−7.66	11.5
	8	Manshui	116.07	31.20	212	−55.6	−8.29	10.7	−49.4	−7.55	10.9
	9	Shiyangm	116.19	31.15	706	−61.3	−9.19	12.2	−56.9	−8.69	12.6
	11	Shiyang	116.21	31.24	217	−58.6	−8.80	11.8	−52.1	−8.05	12.3
	13		116.30	31.25	154	−55.8	−8.53	12.4	−49.8	−7.88	13.2
	14	Maotan	115.99	31.31	260	−56.2	−8.44	11.3	−58.3	−8.75	11.7
	16	Qingtan	116.38	31.17	192	−59.6	−9.09	13.1	−53.3	−8.24	12.6
	19	Saozhou	116.47	31.27	274	−54.8	−8.65	14.4	−50.8	−7.80	11.6
	21		116.35	31.24	123	−61.0	−9.24	13.0	−64.3	−9.59	12.4
	20	Saozhou	116.41	31.25	174	−53.7	−8.39	13.4	−49.2	−7.45	10.4
	23	Kongjia	116.18	31.36	125	−57.9	−9.04	14.4	−51.9	−8.12	13.1
	25	Kongjia	116.24	31.39	94	−57.1	−8.79	13.2	−51.6	−7.95	12.1
	28	Danjiamiao	116.46	31.36	132	−52.4	−8.14	12.7	−47.1	−7.40	12.0
	33	Huangwei	116.32	31.16	198	−60.7	−9.35	14.1	−54.4	−8.03	9.8
	34	Xiongjia	116.36	31.47	48	−50.0	−7.79	12.2	−45.0	−6.40	6.2
Spring water	3		115.78	31.13	974	−68.8	−10.05	11.5	−60.1	−9.05	12.3
	10		116.21	31.21	367	−55.1	−8.36	11.8	−50.9	−8.04	13.4
	17		116.48	31.19	286	−51.4	−8.10	13.4	−46.4	−7.41	12.8
	18		116.47	31.29	366	−55.7	−8.85	15.1	−49.8	−7.99	14.1
	27		116.47	31.31	244	−53.6	−8.39	13.5	−49.9	−8.12	15.1
Reservoir water	2	Xianghongdian	116.00	31.45	122	−58.6	−8.43	8.8	−47.8	−7.18	9.6
	4		115.89	31.24	391	−60.9	−8.66	8.4	−53.9	−8.35	12.9
	5		116.03	31.41	190	−62.8	−8.87	8.2	−59.0	−8.73	10.8
	12	Baiyunya	116.17	31.26	202	−62.0	−9.07	10.6	−52.5	−7.67	8.8
	15		116.09	31.15	376	−62.4	−9.34	12.3	−56.1	−8.42	11.3
	22	Foziling	116.31	31.28	127	−58.8	−8.55	9.6	−53.6	−7.86	9.2
	24	Foziling	116.27	31.34	129	−59.5	−8.80	10.9	−51.9	−7.70	9.7
	26	Banjiezhuizi	116.32	31.44	60	−57.9	−8.81	12.6	−58.5	−8.45	9.2
	32		115.88	31.35	263	−62.4	−9.69	15.1	−58.9	−8.72	10.9
Pond water	29		116.47	31.40	133	−47.5	−6.13	1.6	−40.8	−5.05	−0.4
	30		116.46	31.42	147	−57.9	−8.75	12.1	−44.0	−6.41	7.3
	31		116.46	31.47	113	−52.6	−7.74	9.3	−47.3	−6.08	1.3

* Log.: longitude; Lat.: latitude; Alt.: altitude; *d*-excess: deuterium excess.

**Table 2:** Temperature, humidity, and rainfall in Huoshan, the UPRB [29].

	Max. T	Min. T	Avg. T	Avg. H	Monthly Rainfall	Rainfall Days
	°C	°C	°C	%	mm	Days
October 2017	20.0	12.4	15.5	88.8	180.2	16
September 2018	28.1	19.5	23.0	83.3	128.2	14

* Max. T: mean of the maximum air temperature; Min. T: mean of the minimum air temperature; Avg. T: average air temperature; Avg. H: average relative humidity.

**Table 3:** Maximum, minimum, and average values of different types of water.

Water Types		δ^2^H	δ^18^O	δ^2^H	δ^18^O
		‰	‰	‰	‰
		2017		2018	
River water	Min.	−61.3	−9.35	−64.3	−9.59
	Max.	−50.0	−7.79	−45.0	−6.40
	Avg.	−56.9	−8.67	−52.8	−8.04
Spring water	Min.	−68.8	−10.05	−60.1	−9.05
	Max.	−51.4	−8.10	−46.4	−7.41
	Avg.	−56.9	−8.75	−51.4	−8.12
Reservoir water	Min.	−62.8	−9.69	−59.0	−8.73
	Max.	−57.9	−8.43	−47.8	−7.18
	Avg.	−60.6	−8.91	−54.7	−8.12
Pond water	Min.	−57.9	−8.75	−47.3	−6.41
	Max.	−47.5	−6.13	−40.8	−5.05
	Avg.	−52.7	−7.54	−44.0	−5.85

* Max.: maximum value; Min.: minimum value; Avg.: average value.

**Table 4:** Electrical conductivity (EC), Na%, sodium absorption ratio (SAR), magnesium hazard (MH), and Kelly’s ratio (KR) values of water samples in the UPRB.

Water Types	No.	EC (μs/cm)	Na%	SAR	MH	KR	EC (μs/cm)	Na%	SAR	MH	KR
		2017					2018				
River water	1	128	15.55	6.47	12.82	0.20	124	14.72	6.23	13.61	0.19
	6	99	19.58	7.36	13.14	0.26	117	16.08	6.75	13.24	0.22
	7	131	20.03	8.74	13.94	0.27	213	19.84	7.33	16.52	0.27
	8	166	16.52	7.93	13.08	0.21	208	15.14	6.25	11.42	0.19
	9	60	21.34	5.93	11.60	0.29	62	16.06	6.64	12.82	0.21
	11	96	18.11	6.59	13.63	0.24	112	17.75	5.11	13.78	0.25
	13	84	20.38	6.98	17.73	0.28	98	15.71	6.79	14.36	0.21
	14	171	14.75	7.14	15.86	0.19	116	14.55	6.73	12.66	0.19
	16	72	20.50	6.62	12.33	0.29	120	18.18	6.75	11.13	0.24
	19	154	6.80	2.94	14.86	0.08	170	17.24	7.12	10.91	0.23
	21	80	19.04	6.35	13.12	0.26	58	16.09	6.18	12.57	0.21
	20	151	12.68	5.65	11.99	0.15	176	13.59	5.17	13.70	0.18
	23	113	16.06	6.09	14.76	0.21	136	15.47	5.42	11.85	0.20
	25	136	15.55	6.53	13.37	0.20	158	15.35	6.40	11.98	0.20
	28	163	7.64	3.56	7.83	0.09	214	13.33	4.22	17.91	0.17
	33	111	15.83	6.03	12.70	0.20	101	5.67	2.98	11.90	0.06
	34	218	10.97	6.17	10.36	0.14	298	5.65	2.58	8.82	0.06
Spring water	3	42	12.78	2.94	9.15	0.16	46	19.19	10.83	12.41	0.26
	10	96	20.90	7.83	14.22	0.29	96	6.52	3.11	14.18	0.08
	17	116	22.18	8.79	14.50	0.31	136	15.06	10.13	9.71	0.20
	18	335	1.95	1.18	22.57	0.02	207	14.78	5.69	12.70	0.19
	27	111	5.70	2.08	9.09	0.06	158	15.28	7.18	18.40	0.24
Reservoir water	2	107	14.92	5.69	12.39	0.19	107	17.75	7.41	11.98	0.24
	4	93	18.18	6.59	11.24	0.24	119	16.88	9.29	12.99	0.22
	5	100	15.66	5.81	13.67	0.21	96	21.08	6.67	11.98	0.30
	12	102	16.63	6.33	13.16	0.22	99	12.02	5.97	11.76	0.15
	15	73	19.89	6.43	12.62	0.27	90	7.75	4.03	7.73	0.09
	22	99	15.66	5.71	12.75	0.20	92	18.49	6.65	12.11	0.25
	24	112	15.18	5.85	12.46	0.20	89	15.59	5.26	12.97	0.20
	26	124	15.07	6.17	12.69	0.19	126	16.65	6.16	14.86	0.22
	32	74	19.09	6.02	13.76	0.27	100	21.69	9.62	14.54	0.30
Pond water	29	167	12.79	6.61	15.40	0.19	148	15.84	4.73	16.52	0.21
	30	107	13.23	4.55	17.04	0.17	101	17.13	4.76	7.40	0.24
	31	84	17.81	5.49	14.06	0.24	72	17.82	6.62	12.82	0.24
